# Supplementary material for: A 3.584 Tbps coherent receiver chip on InP-LiNbO3 wafer-level integration platform
Source: Light Sci Appl. 2025 Apr 25;14:172. doi: 10.1038/s41377-025-01821-1 (PMC12032343; doi:10.1038/s41377-025-01821-1)
Supplement: Supplementary file 1 — Supplementary Information [file 41377_2025_1821_MOESM1_ESM.docx]

**Supplementary Information for**

**A 3.584 Tbps coherent receiver chip on InP-LiNbO_3_ wafer-level integration platform**

Xiaojun Xie^1,2,*^, Chao Wei^1,2^, Xingchen He^1^, Yake Chen^1^, Chenghao Wang^1^, Jihui Sun^1^, Lin Jiang^1^, Jia Ye^1^, Xihua Zou^1^, Wei Pan^1^, and Lianshan Yan^1,*^

^1^Key Laboratory of Photonic-Electronic Integration and Communication-Sensing Convergence, School of Information Science and Technology, Southwest Jiaotong University, Chengdu, 611756, China.

^2^These authors contributed equally: Xiaojun Xie, Chao Wei

[^*^](mailto:*)email: xxie@swjtu.edu.cn, [lsyan@home.swjtu.edu.cn](mailto:*lsyan@home.swjtu.edu.cn)

**Supplementary Note 1. Epitaxial layer of the modified uni-traveling carrier photodiode**

Supplementary Table 1 Epi structure of the MUTC photodiode

| **Layer** | **Material** | **Thickness** | **Concentration (cm^-3^)** | **Type** |
| --- | --- | --- | --- | --- |
| N contact   Matching layer | InP | 300 nm | 8 × 10^18^ | N |
| Cladding layer | InP | 100 nm | 5 × 10^18^ | N |
| Sacrificial layer | InP | 40 nm | 1 × 10^18^ | P |
| Drift layer | InP | 120 nm | 3 × 10^16^ | N |
| Cliff layer | InP | 20 nm | 3 × 10^17^ | N |
| Smooth layer | InGaAsP, Q1.1 | 10 nm | 1 × 10^16^ | N |
|  | InGaAsP, Q1.4 | 10 nm | 1 × 10^16^ | N |
| Depleted absorption layer | In_0.53_Ga_0.47_As | 30 nm | 1 × 10^16^ | N |
| Graded doped  absorption layer | In_0.53_Ga_0.47_As | 30 nm | 5 × 10^17^ | P |
|  | In_0.53_Ga_0.47_As | 40 nm | 1 × 10^18^ | P |
|  | In_0.53_Ga_0.47_As | 40 nm | 2 × 10^18^ | P |
| Cap layer | InP | 100 nm | 2 × 10^18^ | P |
| P contact layer | In_0.53_Ga_0.47_As | 50 nm | 2 × 10^19^ | P |
| Buffer layer | InP | 500 nm | 2 × 10^18^ | P |
|  | InP | 350 µm |  |  |

The epitaxial layers of the modified unitraveling carrier photodiode (MUTC-PD) were grown by metal-organic chemical vapor deposition (MOCVD) on a 2-inch semi-insulating InP substrate, as detailed in Supplementary Table 1. Initially, a 50-nm heavily doped (2 × 10^19^ cm^−3^) In_0.53_Ga_0.47_As layer was deposited as the p-contact layer, followed by a 100-nm InP buffer layer. Gradient p-doped In_0.53_Ga_0.47_As absorption layers (ranging from 2 × 10^18^ cm^−3^ to 5 × 10^17^ cm^−3^) were utilized to generate a self-induced electric field, accelerating the diffusion of photogenerated electrons. To mitigate the space charge effect at the In_0.53_Ga_0.47_As/InP interface, a 30-nm n-doped In_0.53_Ga_0.47_As depletion absorption layer, a 20-nm InGaAsP quaternary compound layer, and a 20-nm n-doped InP cliff layer were incorporated. The InGaAsP quaternary compound layer was employed to mitigate the conduction band discontinuity, while the 30-nm n-doped In_0.53_Ga_0.47_As depletion absorption layer and the 20-nm n-doped InP cliff layer (3 × 10^17^ cm^−3^) were used to enhance the electric field, facilitating electron transport. A 120-nm n-doped drift layer (3 × 10^16^ cm^−3^) and a sacrificial 40-nm p-doped InP layer (1 × 10^18^ cm^−3^) were used to optimize the electric field within the drift layer, enabling electron velocity overshoot. A heavily n-doped (8 × 10^18^ cm^−3^) InP layer served as both the n-contact and matching layer. The thickness of the matching layer was optimized as 300 nm to achieve a uniform absorption profile, enhancing the output RF power.


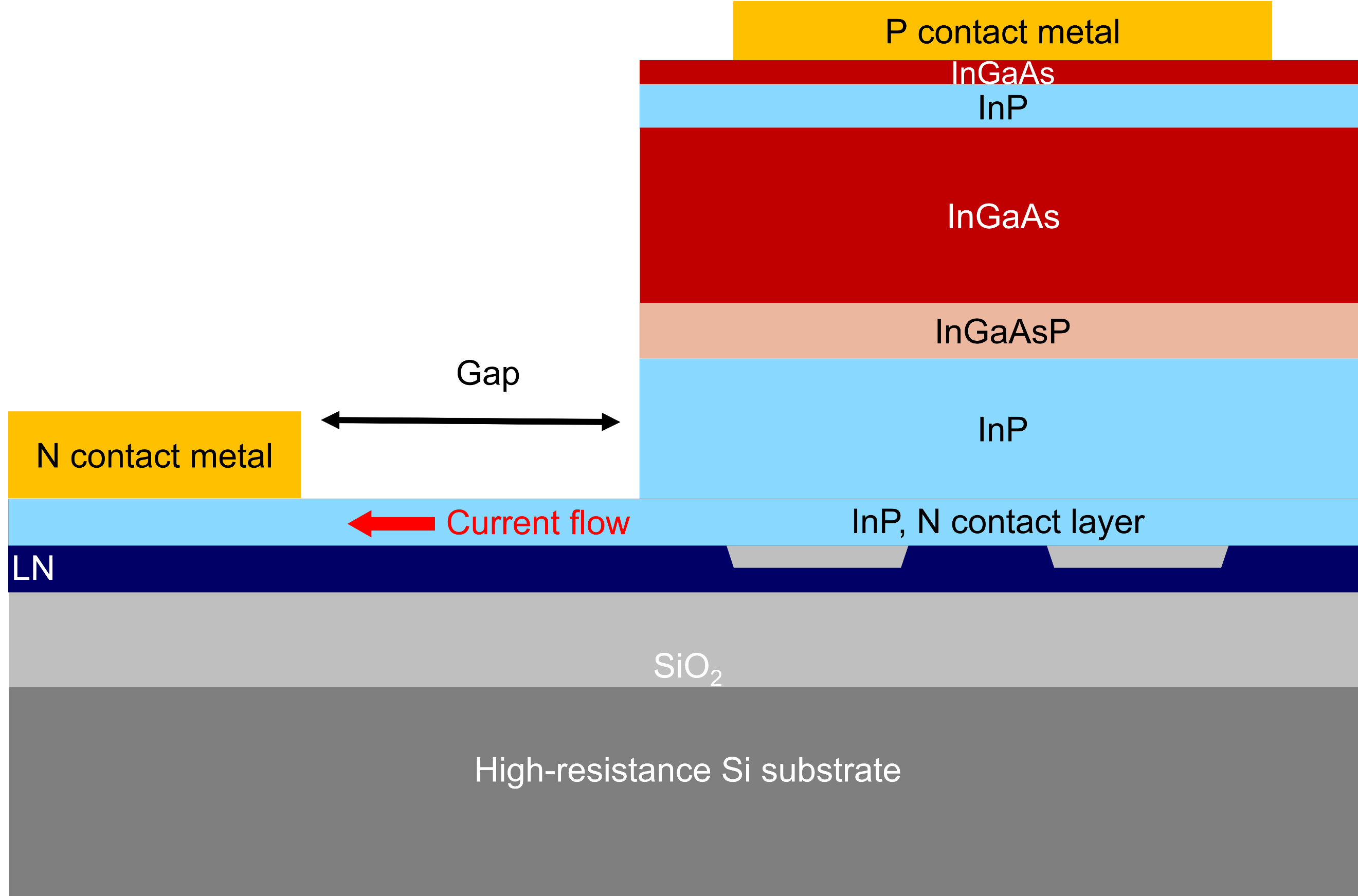


**Fig. S1 Geometry of the heterogeneous integrated photodiode with n-mesa-down structure**.

In our work, this epitaxial structure features a n-mesa-down configuration after wafer bonding to reduce PD series resistance. Fig. S1 shows a schematic plot of the heterogeneous integrated photodiode. The slab InP contact layer is only 300-nm thick so that the cross-section area of the current flow is limited. As a result, the resistance of the slab InP contact layer dominates the resistance of a heterogeneous integrated photodiode. The conductivity of the slab contact layer is the key to achieve a lower resistance. Since the conductivity of the n-doped slab contact layer is higher than p-doped layer by more than 20 times, the n-mesa-down PD structure benefits a low series resistance. A 3D electromagnetic model of the heterogeneous integrated photodiode was configured and the series resistances of the heterogeneous integrated photodiode with n-mesa-down structure and p-mesa-down structure were simulated, which are shown in Fig. S2. It can be observed that the photodiode with n-mesa-down structure has low series resistance compared with the photodiode with p-mesa-down structure. Besides, the photodiode with n-mesa-down structure has a series resistance insensitive to the gap between n contact metal and active mesa, which is friendly to the fabrication process.


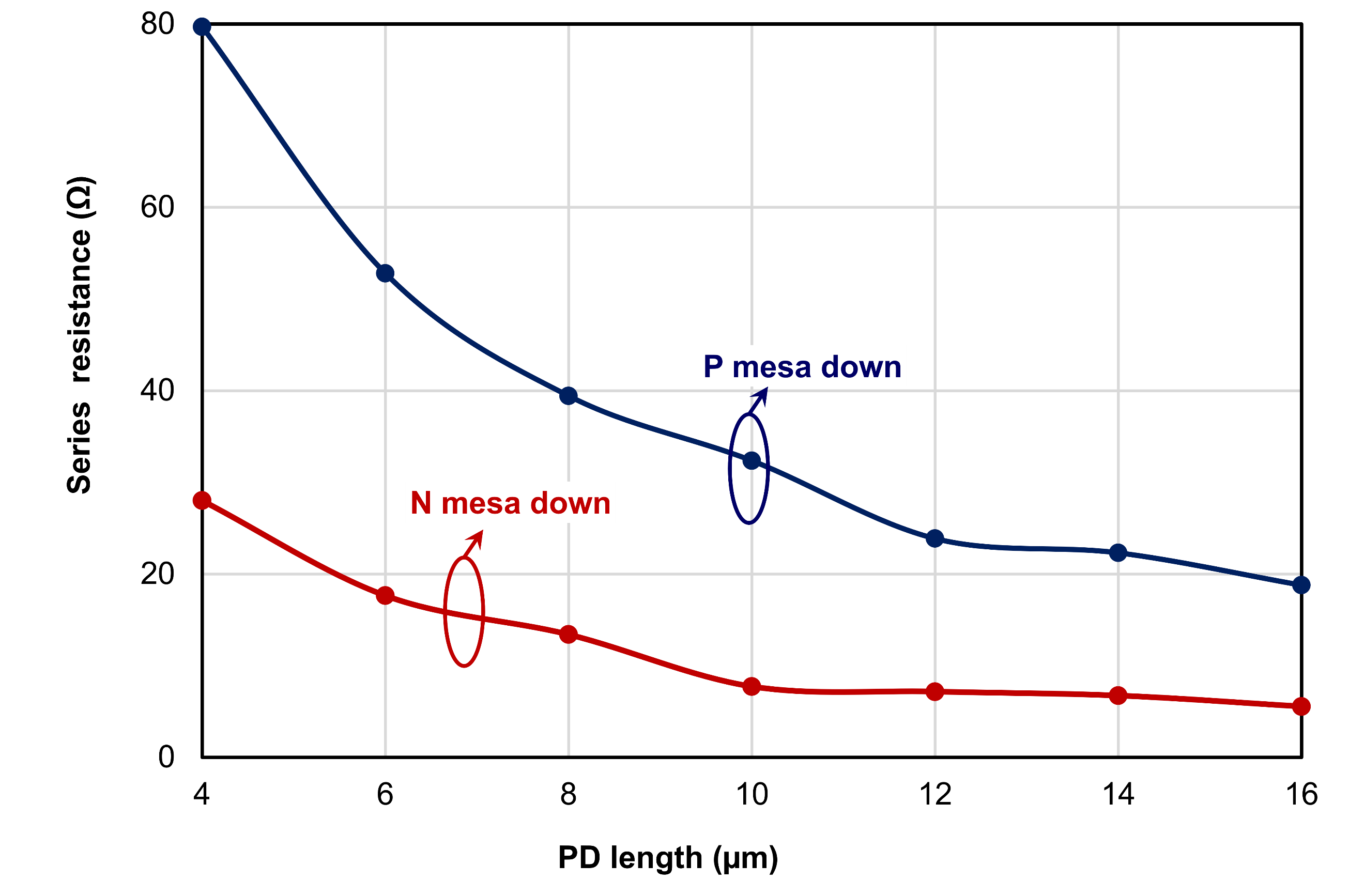


**Fig. S2** Simulated series resistances of the heterogeneous integrated photodiode with n-mesa-down structure and p-mesa-down structure.

**Supplementary Note 2. Dark currents of single photodiodes**

To characterize the yield of the wafer-level InP-LiNbO_3_ heterogeneous integration platform, we randomly selected over 200 single PDs (276 in total) and measured their I-V characteristics. Supplementary Table 2 presents the distribution of dark currents at a -4 V bias voltage. Devices marked with "×" were either short-circuited or disconnected. The majority of devices exhibited dark currents below 300 nA, with some having dark currents as low as a few nA. To calculate the yield, we considered devices functioning properly with a dark current < 1 µA as the qualified devices. The yield was determined to be >80%. Detailed data is provided in Supplementary Table 3. The die-location-dependent yield is shown in Fig. S3. This uniform yield was achieved through a robust wafer-level heterogeneous bonding process and optimized wafer-level fabrication, ensuring uniform performance.

Supplementary Table 2 Summary of dark currents of single PDs

| **Dark Current (nA)** | 0-100 | 100-200 | 200-300 | 300-400 | 400-500 | 500-600 |
| --- | --- | --- | --- | --- | --- | --- |
| **Occurrence** | 58 | 43 | 47 | 26 | 19 | 10 |
| **Dark Current (nA)** | 600-700 | 700-800 | 800-900 | 900-1000 | **>1000** | **×** |
| **Occurrence** | 9 | 2 | 3 | 4 | **45** | **7** |

Supplementary Table 3 Measured dark currents of single PDs (unit: nA)

| 5.24 | 1.62 | 2.52 | 532.17 | 3.35 | 3.60 | 135.30 | 6.74 |
| --- | --- | --- | --- | --- | --- | --- | --- |
| 1713.21 | 834.68 | 16.64 | 3.01 | 4.75 | 3.17 | 4.93 | 14618.32 |
| 216.13 | 124.59 | 174.06 | 255.81 | 212.68 | 382.80 | 223.53 | 258.02 |
| 391.46 | 140.41 | 0.95 | 181.57 | 116.72 | 125.00 | 161.67 | 94.57 |
| 154.75 | 15.17 | 4.19 | 23.17 | 155.85 | 2616.15 | 623.10 | 88.81 |
| 24.44 | 24479.02 | 9162.94 | 2317.67 | 5.14 | 6.59 | 3.73 | 20415.20 |
| 448.47 | 644.30 | × | 108.88 | 159.19 | 233.54 | 277.09 | 235.61 |
| 72.67 | 553.25 | 222.67 | 95.92 | 542.99 | 435.03 | 184.40 | 347.08 |
| 3583.93 | 4.12 | 4193.39 | 2120.24 | 333.74 | × | 25.15 | 3.11 |
| 382.61 | × | 1394.95 | 87.71 | 551.46 | 99.43 | 451.32 | 911.68 |
| 205.09 | 116.50 | 297.85 | 82.88 | 391.09 | 412.67 | 173.81 | 515.44 |
| 229.60 | 157.28 | 126.62 | 0.80 | 69.93 | 347.27 | 251.95 | 250.31 |
| 48.33 | 11721.18 | 26.13 | 99.17 | 69.24 | 1883.67 | 109.66 | × |
| 174.54 | 471.63 | 634.91 | 23.95 | 237.20 | 2438.89 | 263.11 | 4512.69 |
| 421.42 | 378.15 | 501.84 | 250.44 | 531.00 | 307.04 | 365.19 | 304.69 |
| 134.09 | 366.40 | 223.95 | 285.07 | 364.06 | 381.61 | 287.18 | 604.61 |
| 4529.31 | 1359.84 | 39.16 | 1178.48 | 6012.16 | × | 1561.77 | 7031.42 |
| 200.00 | 5715.49 | 176.52 | 5489.14 | 228.33 | 6638.08 | 647.08 | 1388.96 |
| 279.72 | 146.39 | 395.54 | 256.64 | 290.24 | 262.72 | 468.01 | 123.66 |
| 239.56 | 1220.85 | 236.56 | 562.43 | 471.20 | 551.30 | 400.45 | 483.69 |
| 1069.59 | 31.73 | 6232.82 | 24.53 | 741.42 | 9626.40 | 617.77 | 31902.06 |
| 334.64 | 255.61 | 172.51 | 323.04 | 194.80 | 278.89 | 157.12 | 197.55 |
| 762.01 | 457.45 | 673.34 | 496.37 | 4.64 | 6.11 | 6.54 | 153.54 |
| 9.66 | 57.47 | 40.07 | 36.72 | 205.87 | 398.28 | 145.76 | 338.93 |
| 119.14 | 5.94 | 31.16 | 1714.99 | 100.11 | 339.03 | 198.31 | 293.67 |
| 205.26 | 434.51 | 202.71 | 190.80 | 5309.90 | 3748.39 | 999.55 | 4595.70 |
| 86.47 | 266.02 | 235.63 | 132.91 | 15.56 | 6186.40 | 249.25 | 33984.15 |
| 18757.35 | × | 103.84 | 105.08 | 1150.41 | 161.87 | 495.07 | 362.46 |
| 69.41 | 971.10 | 18.54 | 2746.82 | 595.82 | 462.57 | × | 17.84 |
| 127.66 | 243.30 | 102.70 | 299.75 | 665.56 | 2877.88 | 2362.49 | 299.75 |
| 669.23 | 291.21 | 70.47 | 145.98 | 399.51 | 16.71 | 114.31 | 16.51 |
| 113.34 | 2497.25 | 6841.85 | 7.09 | 166.24 | 367.48 | 475.78 | 422.76 |
| 27.87 | 12.13 | 258.06 | 9.77 | 255.09 | 231.61 | 245.30 | 492.47 |
| 239.62 | 860.21 | 264.34 | 1278.99 | 1577.02 | 973.17 | 815.27 | 17.72 |
| 304.64 | 343.97 | 414.77 | 322.26 |  |  |  |  |

Devices marked with "×" were either short-circuited or disconnected.

**
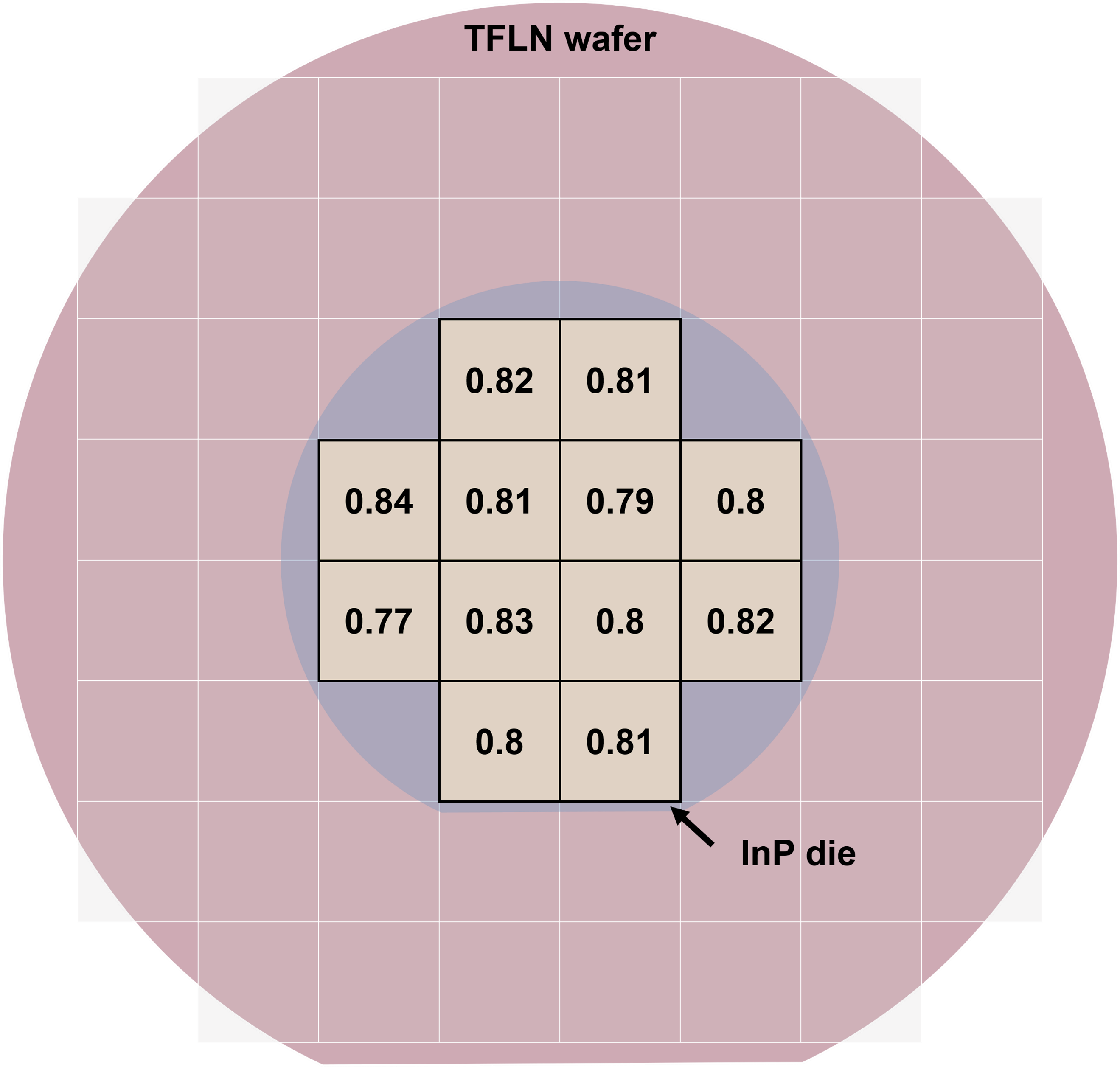
**

**Fig. S3** The yield across the 2-inch PD region on the 4-inch LN wafer.

**Supplementary Note 3. High-speed electro-optic** **intensity modulator based on the InP-LiNbO_3_ wafer-level integration platform.**


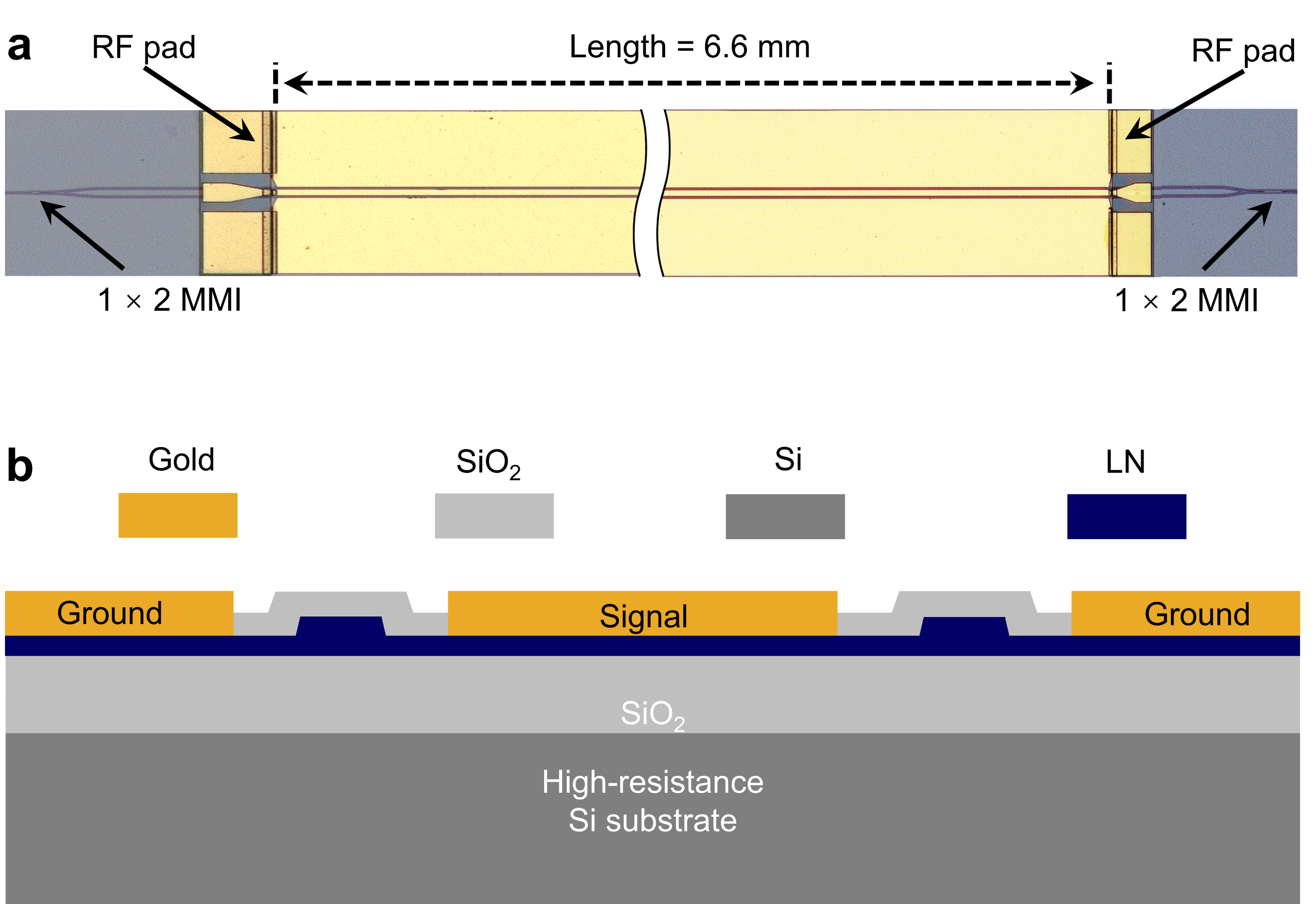


**Fig. S4** **Electro-optic LN intensity modulator based on InP-LiNbO_3_ wafer-level integration platform**. **a** Microscope image and **b** cross-section of the modulator.

Fig. S4**a** presents a microscope image of the electro-optic modulator fabricated on an InP-LiNbO₃ wafer-level integration platform. The intensity modulator adopts a ground-signal-ground (GSG) traveling-wave design implemented on an x-cut thin-film LN-on-insulator platform. The input light is split into two arms of a Mach-Zehnder interferometer (MZI) based on a 1×2 multi-mode interferometers (MMI) coupler, where the light co-propagates with a microwave drive signal along a GSG transmission line electrode. Fig. S4**b** illustrates the cross-sectional structure of the modulation region. The traveling microwave signal modulates the optical signal over the electrode in a push-pull configuration, where an optical phase advance in one arm and an optical phase delay in the other. A 50-Ω GSG pads is used to input and output RF signals. The width of the optical waveguide is set as 1.5 µm. As to the geometry of the transmission line electrode, the width and length of the signal trace is 17 µm and 6.6 mm, respectively. The gap between the signal trace and the ground trace is 6 µm as a trade-off between optical loss and modulation efficiency. The optical waveguides and the transmission line electrode are precisely defined on wafer-level scale by an i-line stepper lithography system. It should be noted that the fabrication process of the modulator is carried out after finishing the photodiode process and all photodiode area is protected during the modulator process. Therefore, our InP-LiNbO_3_ heterogeneous integration platform has the capability to integrate the transmitter and receiver on the same LN chip. Beyond the high-performance transceiver for optical communication, the multi-function InP-LiNbO_3_ wafer-level heterogeneous integration platform has the potential to achieve full-photonics THz communication and sensing integrated chips, integrated adaptive coherent LiDAR chips, and large-scale integrated photonics computing chips.


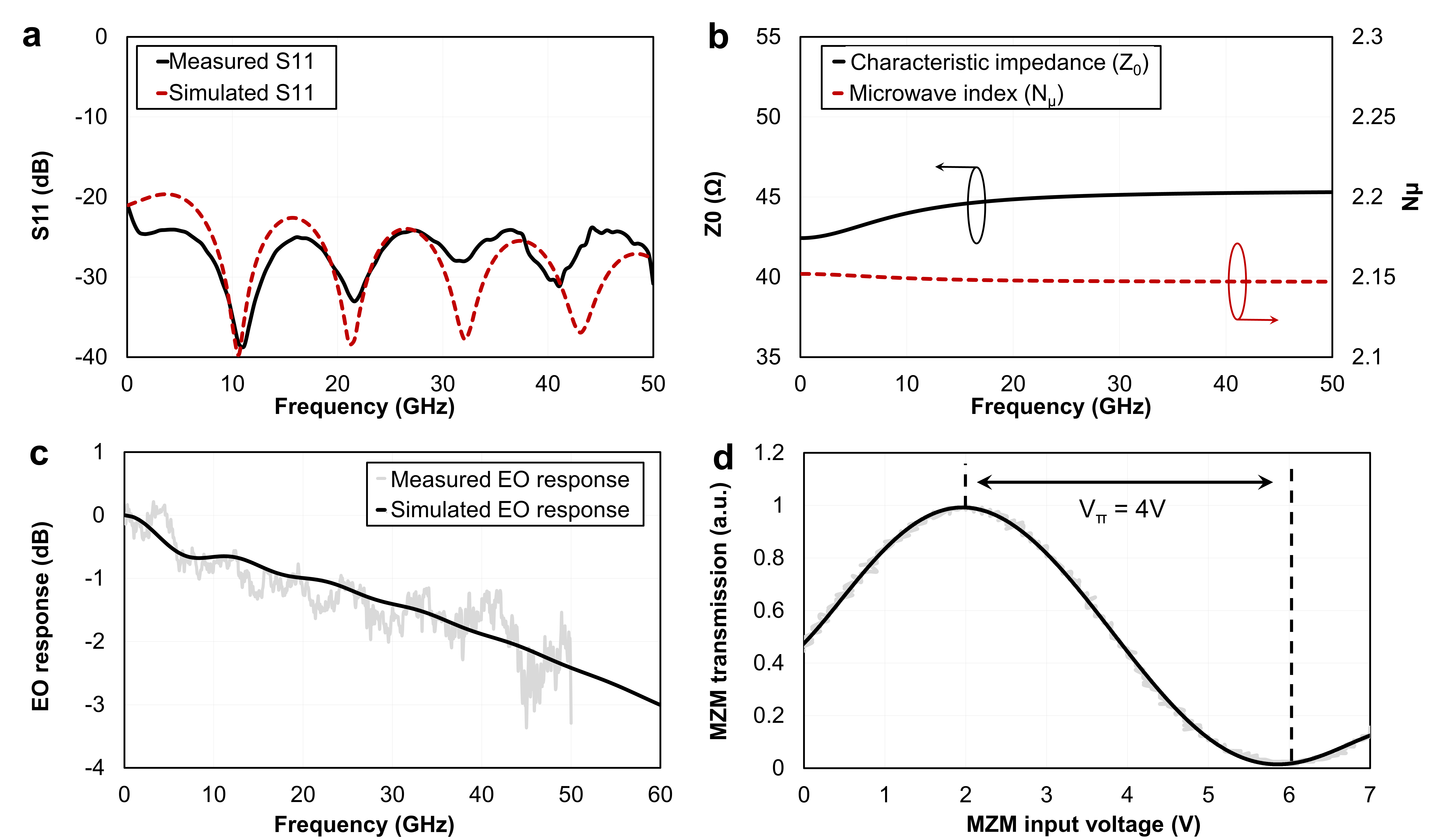


**Fig. S5** **Electro-optic performance of the electro-optic LN modulator.** **a** Measured and simulated electrical reflection (S_11_). **b** Extracted characteristic impedance and microwave effective index of the transmission line. **c** Measured and simulated EO response. **d** Measured voltage power curve of the modulator.

The electro-optic performance of the fabricated modulator is characterized comprehensively, including electrical reflection (S_11_), characteristic impedance (Z_0_), microwave effective index (N_µ_), EO response (3-dB bandwidth), and half-wave voltage (V_π_). The S_11_ was measured by a vector network analyzer (VNA), as shown in Fig. S5**a**. A comprehensive theoretical model was developed for analyzing the EE and EO frequency response of traveling-wave optical modulators^1^. Based on the S_11_ fitting, the extracted capacitance and inductance of the transmission line is 157.6 fF mm^-1^ and 325 pH mm^-1^, respectively. As shown in Fig. S5**b**, the corresponding characteristic impedance and microwave effective index is 45 Ω and 2.15, respectively. Fig. S5**c** shows the measured and simulated EO response with 50 Ω termination and the 3-dB EO bandwidth of the modulator is > 50 GHz. With a low-frequency bias voltage applied to the modulator, the transmission curve of the modulator can be obtained, shown in Fig. S5**d**. It can be observed that V_π_ of the modulator is 4 V, corresponding to a modulation efficiency of 2.64 V·cm.

**Supplementary Note 4. Experimental setup to measure bandwidth and output power of photodiodes**

**
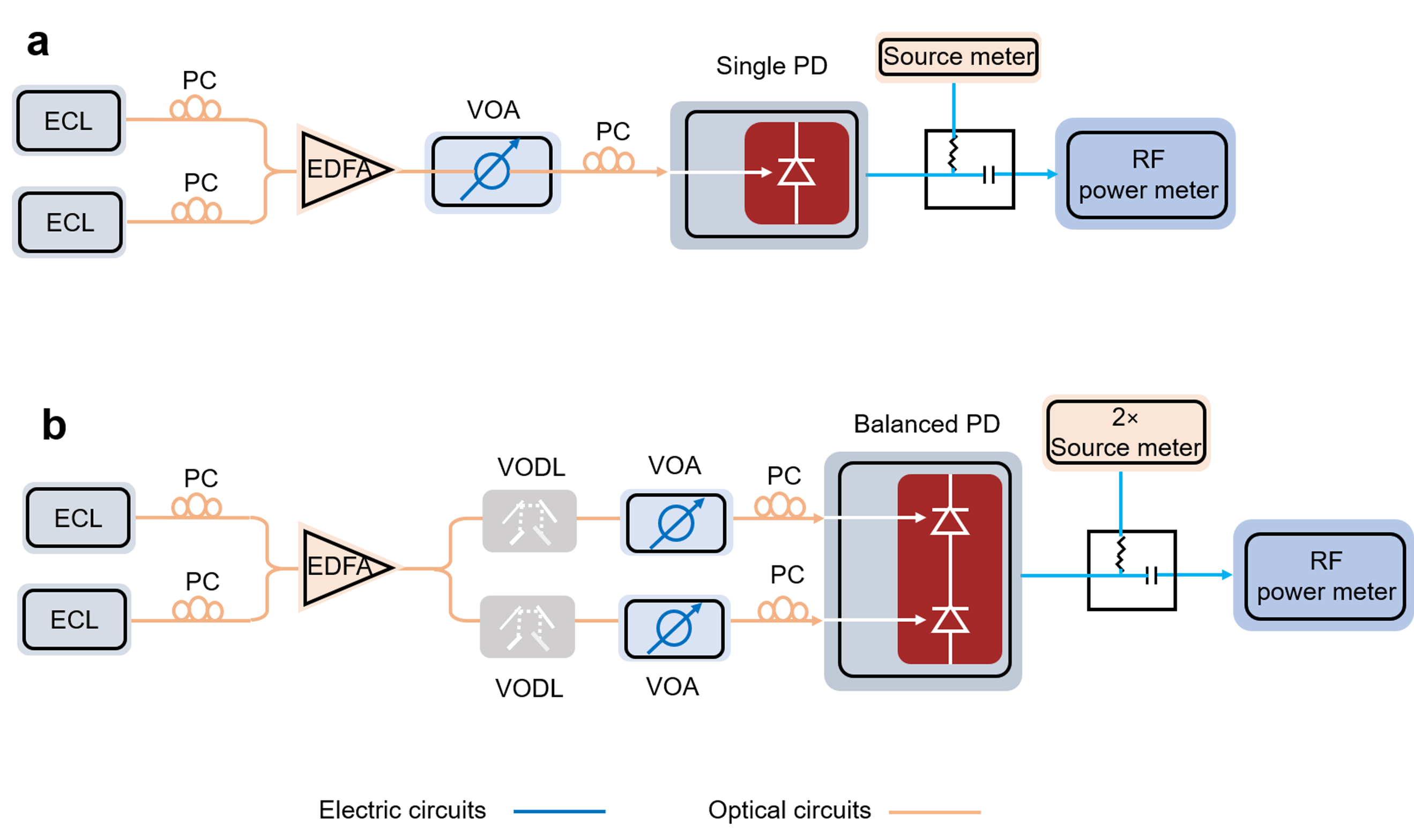
**

**Fig. S6 Frequency response measurement setup**. **a** Experimental setup for single PD and PD array, **b** Experimental setup for balanced PD. ECL: external cavity laser, PC: polarization controller, EDFA: erbium-doped fiber amplifier, VOA: variable optical attenuator, VODL: variable optical delay line, RF power meter: radio frequency power meter.

The schematic diagram for measuring the bandwidth of the single PD and PD array using the heterodyne method is shown in Fig. S6**a**. Two continuous wave (CW) lights from ECLs (Keysight 81940A) were combined using a 3-dB coupler to generate a beat signal with 100% modulation depth. The frequency of the beat signal was determined by the frequency difference between the two CW lights. The optical power was amplified by a high-power EDFA. The amplified light was coupled into the LiNbO_3_ waveguide using a lensed fiber with a 2.5-μm spot diameter and subsequently detected by the single PD. The overall frequency response of the device was measured using ground-signal-ground (GSG) probes covering DC–67 GHz, 75 GHz–110 GHz, 90 GHz–140 GHz, and 110 GHz–170 GHz frequency bands. In addition to bandwidth measurement, the saturation behavior of the single PD and PD array was characterized by the same heterodyne setup. By fixing the beat frequency and adjusting optical attenuators to change the incident light power, photocurrents and the corresponding radio frequency (RF) output power were obtained.

Fig. S6**b** illustrates the frequency response measurement setup for the balanced PD. Two CW lights from ECLs were combined using a 3-dB coupler to generate a beat signal with 100% modulation depth. The optical beat signals were split into two paths, each incorporating a variable optical delay line (VODL) to control the phase difference between the two signals. Common-mode (even multiples of π phase difference) and differential-mode (odd multiples of π phase difference) were obtained by tuning the phase difference. Variable attenuators were used to compensate for power imbalances caused by losses in the two optical paths. Finally, the optical signals from each path were coupled to the balanced PD via lensed fibers. A DC–67 GHz RF probe was used to measure the bandwidth of the balanced PD.

**Supplementary Note 5. Optical coupling between LN waveguide and PD active layers.**


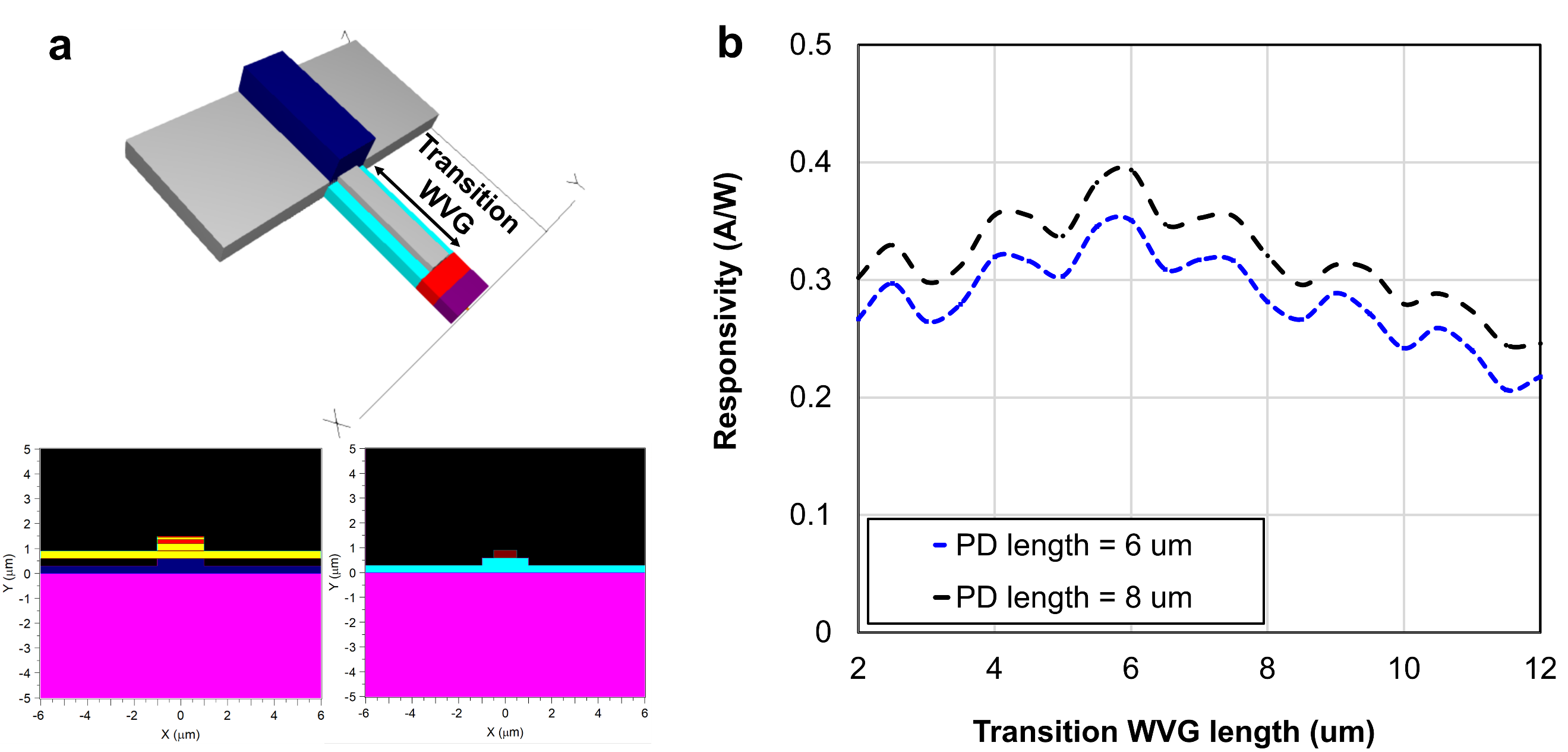


**Fig. S7** **PD responsivity simulation**. **a** 3-D geometry and cross-section of the integrated PD. **b** Simulated responsivity with different transition waveguide lengths.

The InP photodiodes were bonded onto the thin-film lithium niobate waveguides directly. The evanescent field of the optical mode in the waveguide overlaps with the InGaAs absorption layers of the InP photodiode, enabling efficient light transfer. Since the PD structure with n-mesa-down configuration was applied to reduce series resistance, the waveguide was separated from InGaAs absorption layers by InP drift layers so that the evanescent coupling was relative weak compared with p-mesa-down structure. In this work, we adopted a InP transition waveguide to improve the responsivity. The simulated responsivity with different transition waveguide length is presented in Fig. S7**b**. It can be observed that the responsivity ripples with the transition waveguide due the constructive and destructive interference between the transition waveguide and absorption layers. The simulated responsivity reached 0.4 A W^-1^ for an 8-µm-long PD with 6-µm-long transition waveguide, which agrees with the measured responsivity. In order to improve the responsivity, further optimization is required. One option is to incorporate adiabatic transition waveguide to reduce the mode mismatch between InP transition waveguide and LN waveguide. The expected responsivity is close to 0.7 A W^-1^ for the same length photodiode.

**Supplementary Note 6. Equivalent circuit model and S-parameter of the single photodiode**

**
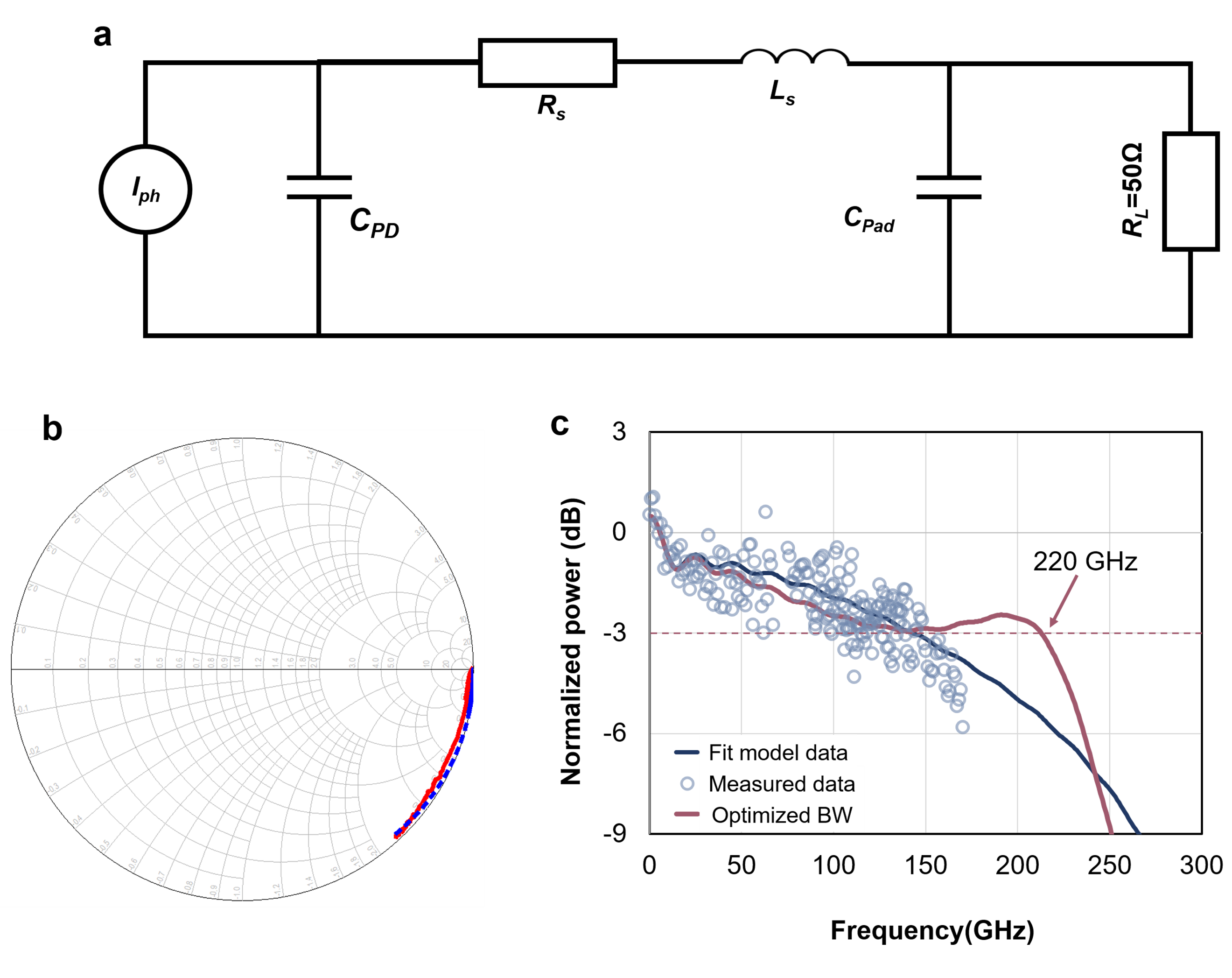
**

**Fig. S8 equivalent circuit model and S-parameter of the single PD**. **a** Equivalent circuit model of the single PD for S_11_ fitting and frequency response simulation. *I_ph_*: current source, *C_PD_*: junction capacitance, *R_s_*: series resistance, *C_pad_*: CPW capacitance, and *L_s_*: CPW inductance. **b** Measured (red solid line) and fitted (blue dash line) S_11_ data (frequency range: 1 GHz to 67 GHz). **c** Measured (grey circle), fitted (black solid line), and optimized (purple solid line) frequency response.

The scattering parameter S_11_ of the single PD was measured using a network analyzer (Ceyear 3672E) with a scanning frequency of DC–67 GHz. By establishing the equivalent circuit model and fitting the parameters, we extracted physical parameters of the device. As illustrated in Fig. S8**a**, the equivalent circuit model consists of a current source and various RC elements. Specifically, *C_PD_*, *R_s_*, *C_pad_*, and *L_s_* represent the junction capacitance (including parasitic capacitance), series resistance, capacitance and inductance of the coplanar waveguide pad, respectively. The current source is frequency-dependent and expressed as follow

$I_{ph}(f)=I_{0}\left[\frac{1-e^{j\cdot2\pi\cdot t_{r}\cdot f}}{j\cdot2\pi\cdot t_{r}\cdot f}\right]$, (1)

where *f*, *I*_0_, and *t_r_* denote the frequency, DC photocurrent, and electron transit time, respectively.

Figure S8**b** shows the measured and fitted S_11_ on Smith charts at -4 V bias voltage. The extracted capacitance, and resistance were 11.5 fF and 12 Ω, respectively. The ideal junction capacitance can be calculated by a simple analytical equation:

$C_{pn}=\frac{\varepsilon_{0}\varepsilon_{r}S}{D}$, (2)

where *ε_0_* and *ε_r_* represents the permittivity of free space, the dielectric constant of InP, respectively. *S* and *D* is the active area of the device (1.5 μm × 8 μm) and the thickness of the depleted region (230 nm), respectively. The calculated ideal capacitance was 5.5 fF, indicating a parasitic capacitance of 6 fF. The pad capacitance and inductance were estimated as 8.5 fF and 42 pH, respectively. By optimizing the pad capacitance as 19.5 fF and inductance as 65 pH, the 3-dB bandwidth could be increased to 220 GHz, as shown by the purple solid line in Supplementary Fig. S8**c**.

**Supplementary Note 7. Bandwidth of the 1×4 photodiode array**

**
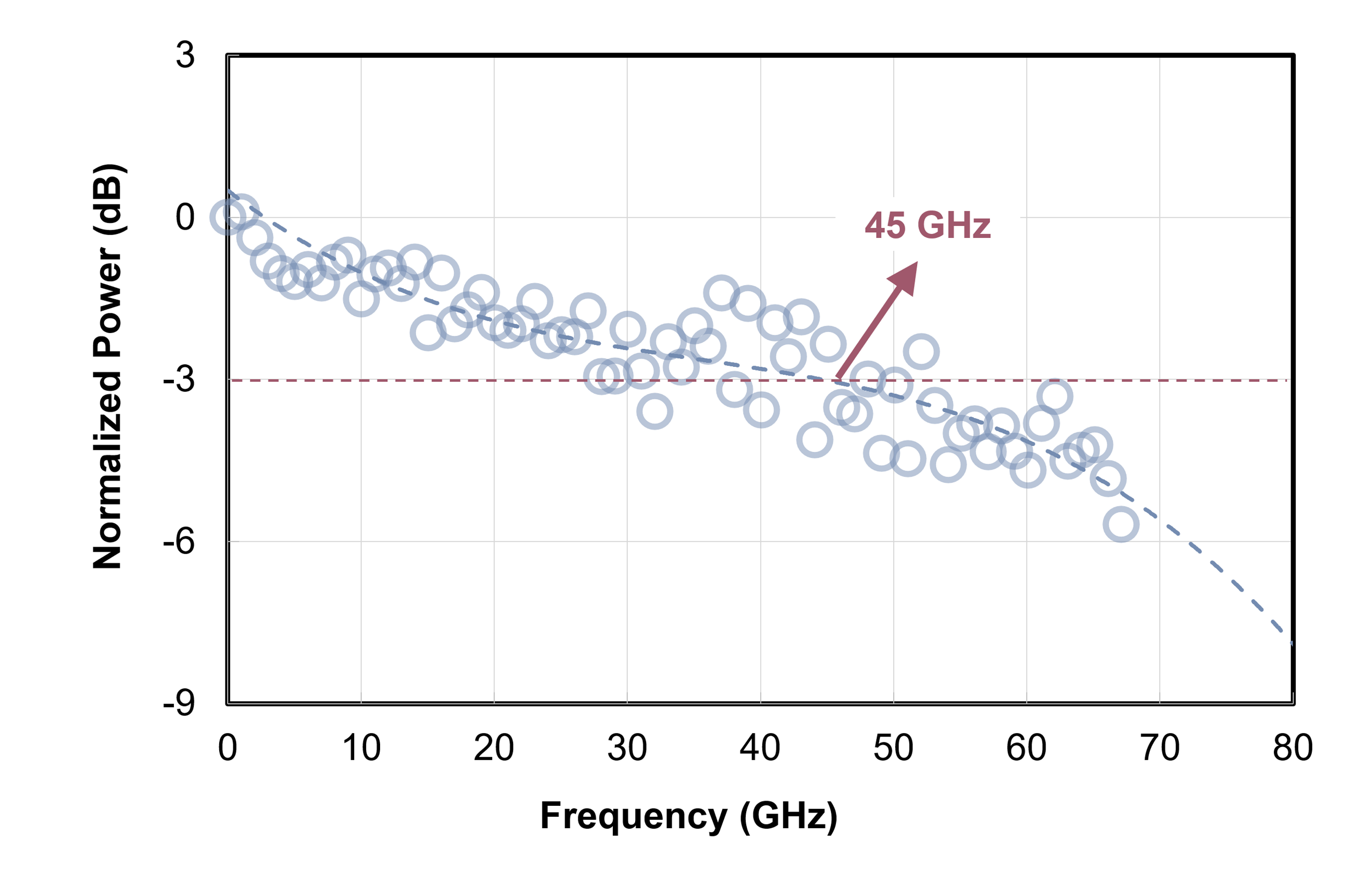
**

**Fig. S9** OE frequency response of 1×4 PD array, grey circle: measured result, blue dash line: fitted curve.

The bandwidth of a 1×4 PD array was measured using the experimental setup shown in Fig. S6**a**. The initial wavelength of both lasers was fixed at 1550 nm. A beat signal with varying frequency was obtained by changing the wavelength of one laser with 1 GHz tuning step. The device exhibited a 3-dB bandwidth of 45GHz (Fig. S9).

**Supplementary Note 8. Design and characterization of optical 90° hybrid coupler**

**
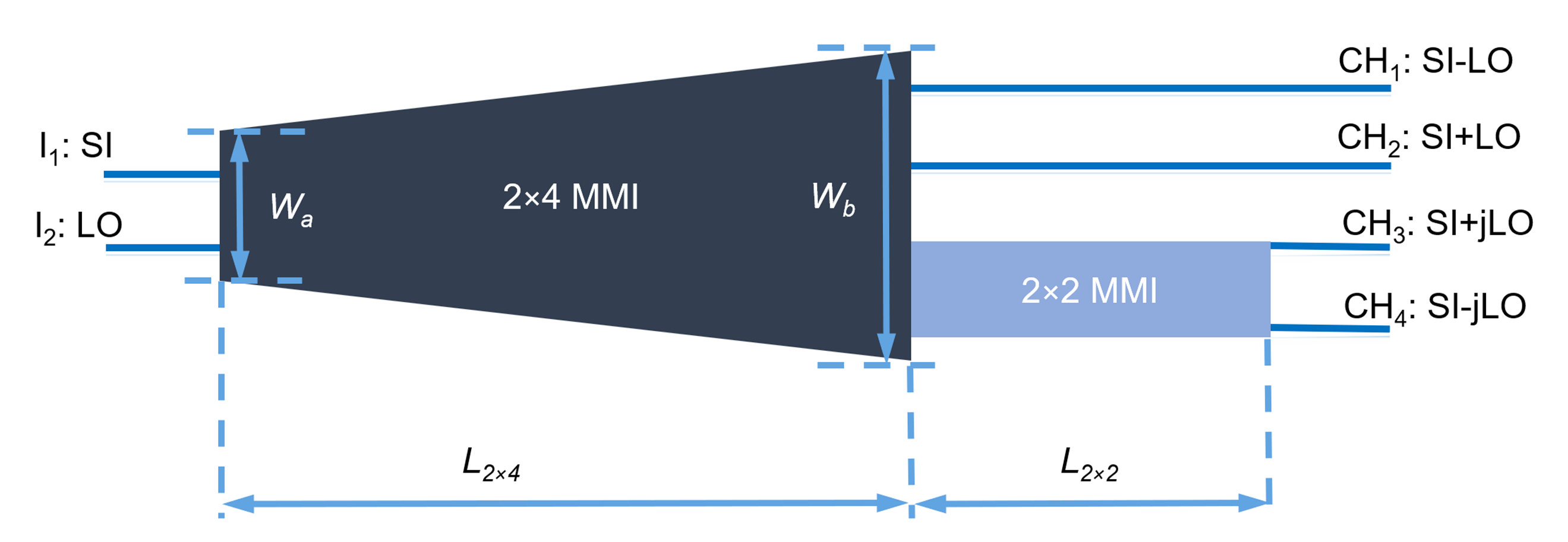
**

**Fig. S10** Structure diagram of the optical 90° hybrid coupler.

The optical 90° hybrid coupler consists of a 2 × 4 MMI coupler based on paired interference and a 2 × 2 MMI coupler based on general interference, as illustrated in Fig. S10. To minimize the MMI size, the 2 × 4 MMI employs a wedge structure. Due to the symmetry of this structure, the two adjacent outputs of the 2 × 4 wedge-shaped MMI exhibit an in-phase relationship. For coherent reception, a 2 × 2 MMI coupler based on general interference is used to adjust the phase relationship of a pair of outputs by 90°.

Without considering the Goos-Hahnchen effect, the MMI beat length ($L_{\pi}$) is defined as:

$L_{\pi}=\frac{\pi}{\beta_{0}-\beta_{1}}$ (3)

where $\beta_{0}$ and $\beta_{1}$ denote the propagation constant of the fundamental mode and the first order mode, respectively. In a 2 × 4 wedge-shaped MMI coupler, the first 4-fold image appears at the MMI region length of:

$L_{2\times4}^{rec}=\frac{L_{\pi}}{4}$ (4)

For the 2 × 4 wedge-shaped MMI coupler, χ is defined the ratio between $W_{a}$ and $W_{\text{b}}$. The MMI beat length ($L_{\pi}^{WS}$) and length ($L_{2\times4}^{WS}$) can be expressed as:

$L_{\pi}^{WS}=L_{\pi}\cdot\chi$ (5)

$L_{2\times4}^{WS}=\frac{L_{\pi}^{WS}}{4}=\frac{L_{\pi}\cdot\chi}{4}$ (6)

The wavefront bending caused by the geometry of the wedge-shaped MMI determines the relative phase difference between each output port of the 2 × 4 wedge-shaped MMI coupler. The MMI coupler divides the optical power into each output channel in a discrete phase relationship. The output phases in a paired-interference-based 2 × 4 wedge-shaped MMI coupler () are given by:

(7)

(8)

where and is the phase of the output port *b* with input port 1 and port 2, respectively. $\eta$= 0 when the output port is odd and $\eta$= 1 for the even-numbered output port. From Equations (7) and (8), it can be seen that CH_3_ and CH_4_ have an inherent phase difference of -45$^{\circ}$. Based on this, the relationship between $W_{a}$ and $W_{\text{b}}$ can be optimized to provide an extra phase difference of -45$^{\circ}$, which achieves phase matching between the 2 × 4 wedge-shaped MMI coupler and the 2 × 2 MMI coupler. This method avoids the use of phase shifter and reduces device complexity. Therefore, the relative phase relation between CH_3_ and CH_4_ of the 2 × 4 wedge-shaped MMI coupler ($\Delta\theta_{34}$) can be expressed as

(9)

where and $\Delta p_{34}$represent the inherent phase difference and the introduced additional phase difference between CH_3_ and CH_4_ of the MMI coupler, respectively.

The width $W_{\text{b}}$ is fixed at 18 μm. The relationship between phase difference and χ can be obtained using Lumerical finite difference time domain (FDTD) simulation. When the χ-value is 0.5, the phase difference between the 2 × 4 wedge MMI coupler CH_3_ and CH_4_ is −90°, and the phase matching between the 2 × 4 MMI coupler and the 2 × 2 MMI coupler is realized.

The schematic diagram for measuring the phase deviation, excess loss and CMRR of the 90° optical hybrid coupler is shown in Fig. 11. The experimental setup includes a tunable laser, a polarization controller (PC), a lensed fiber with a 2.5 µm spot size, and an optical power meter (Keysight N7745A). The tunable laser was adjusted to operate within a wavelength range of 1530 to 1560 nm. The polarization state of the light beam was carefully tuned to the TE mode using the PC. The lensed fibers were precisely aligned with the input and output waveguides using a five-dimensional coupling system. The output light was monitored by the power meter.


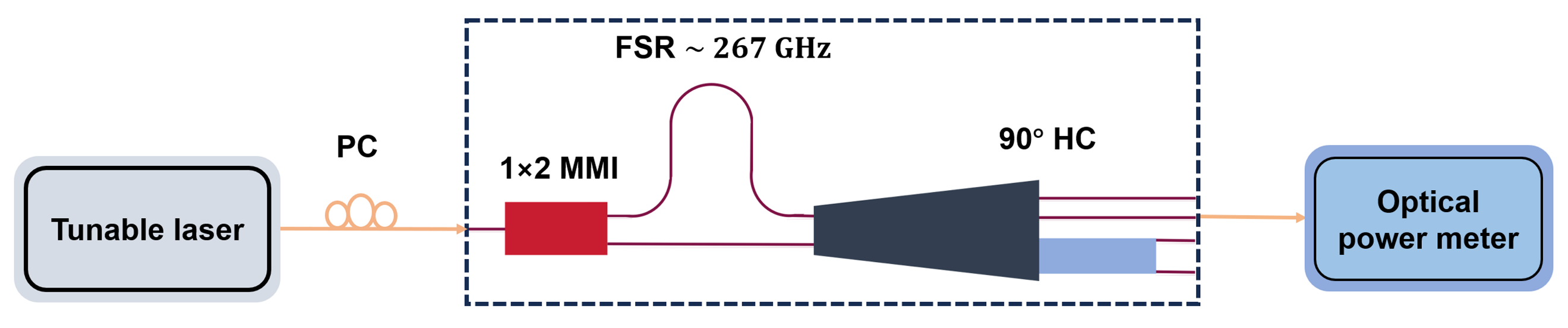


**Fig. S11** Experimental setup of 90°hybrid coupler. PC: polarization controller, MMI: multimode interference coupler, FSR: free spectral range, HC: hybrid coupler

The phase difference between the four outputs of the fabricated device can be measured using a Mach–Zehnder interferometer (MZI). The MZI delay line results in a transmission spectrum period of 267 GHz, which corresponds to a free spectral range (FSR) of approximately 2.14 nm at a wavelength of 1550 nm. Figure 3f of the manuscript shows the measured transmission curves of the fabricated device. A π relative phase difference between the in-phase channels (CH-1 and CH-2) and the quadrature channels (CH-3 and CH-4) was observed. An extinction ratio exceeding 20 dB was achieved over the spectral range of 1530 nm to 1560 nm. Figure 3g of the manuscript shows that the phase deviation of the device is less than ±5°. Figure 3h of the manuscript shows that the device's excess loss remains below 0.5 dB across the same spectral range. As shown in Fig.3i of the manuscript, the CMRR of the corresponding I/Q channels is greater than 20 dB across the wavelength span of 1530 nm to 1560 nm.

**Supplementary Note 9. Summary of energy consumption of different signals.**

To better evaluate the performance of the device in a high-speed coherent communication system, we characterized the energy consumption of receiving advanced modulated signals at different baud rates. The results are presented in Supplementary Table 4. The energy consumption is calculated using the following expression:

$EC=\frac{P_{LO}+ P_{signal}+P_{PA}+4\times I_{ph}\times V_{bias}}{C}$ (10)

where$P_{LO}$, $P_{signal}$,$P_{PA}$, $I_{ph}$, $V_{bias}$, and $C$ represents the received local light power, signal light power, RF amplifier power consumption, photocurrent, applied bias voltage, and capacity, respectively. Since no RF amplifier was required, the coherent receiver exhibited low energy consumption for signals with high-rate and advanced modulation formats. The coherent receiver achieved an ultra-large communication capacity and exhibited an ultra-low energy consumption as low as 9.6 fJ bit^-1^ for 200 Gbit s^-1^ capacity and 13.5 fJ bit^-1^ for 400 Gbit s^-1^ capacity.

Supplementary Table 4 Energy consumption of advanced modulated signals

| **Signal** | **LO**  **Power (mW)** | **Signal**  **Power**  **(mW)** | **Photocurrent**  **(mA)** | **Bias Voltage**  **(V)** | **Power**  **Consumption**  **(mW)** | **Capacity (Gbit s^-1^)** | **Energy**  **Consumption**  **(fJ bit^-1^)** |
| --- | --- | --- | --- | --- | --- | --- | --- |
| 100 Gbaud QPSK | 0.63 | 0.1 | 0.07 | 4 | 1.92 | 200 | 9.59 |
| 100 Gbaud 16 QAM | 1.78 | 0.28 | 0.21 | 4 | 5.38 | 400 | 13.45 |
| 128 Gbaud 16 QAM | 4.47 | 0.71 | 0.56 | 4 | 14.13 | 512 | 27.61 |
| 100 Gbaud 32 QAM | 4.47 | 0.71 | 0.58 | 4 | 14.37 | 500 | 28.75 |
| 80 Gbaud 64 QAM | 4.47 | 0.71 | 0.52 | 4 | 13.53 | 480 | 28.20 |
| 100 Gbaud 64 QAM | 10 | 2 | 0.8 | 4 | 24.8 | 600 | 41.33 |

References

1. Kharel, P., Reimer, C., Luke, K., He, L. & Zhang, M. Breaking voltage–bandwidth limits in integrated lithium niobate modulators using micro-structured electrodes. *Optica* **8**, 357–363 (2021).
